# New approaches to idiopathic neutropenia in the era of clonal hematopoiesis

**DOI:** 10.1186/s40164-023-00403-4

**Published:** 2023-04-28

**Authors:** Olisaemeka D. Ogbue, Tariq Kewan, Waled S. Bahaj, Carmelo Gurnari, Valeria Visconte, Jaroslaw P. Maciejewski

**Affiliations:** 1 Department of Translational Hematology and Oncology Research, Taussig Cancer Institute, 9620 Carnegie Ave N Building, Building NE6-250, Cleveland, OH 44106 USA; 2 Department of Hematology and Oncology, Yale University, New Haven, CT USA; 3 Division of Medical Oncology & Hematology, School of Medicine, University of Louisville, Louisville, KY USA; 4 Department of Biomedicine and Prevention, Molecular Medicine and Applied Biotechnology, University of Rome Tor Vergata, Rome, Italy

**Keywords:** Neutropenia, T-cell mediated, Clonal hematopoiesis

## Abstract

Isolated chronic idiopathic neutropenia (CIN) is a rare disease with multiple contributing etiologies that must be ruled out before establishing a diagnosis. We studied clinical and molecular data of 238 consecutive adult patients with CIN. Autoimmune neutropenia was present in 28% of our cohort. In contrast, T cell-mediated neutropenia was the main underlying pathological mechanism among patients with T cell expansions, such as T-cell large granular lymphocytic leukemia (T-LGL) and T cell clonopathy of undetermined significance, found in 37% and 8% of cases, respectively. Patients with neutropenia also had hypogammaglobulinemia (6%) and/or monoclonal gammopathy of undetermined significance (5%). NGS application has further broadened the spectrum of causes of CIN by including manifestations of clonal hematopoiesis, present in 12% of cases. *TET2* (3%), *TP53* (2%), and *IDH1/IDH2* (2%) mutations were the most commonly found and were enriched in cases with T-LGL. We show that these clinico-molecular associations can be simultaneously present, complicating a proper diagnostic distinction within the broader entity of seemingly idiopathic neutropenia of autoimmune origin. Identification of etiologic culprits may also guide rational selection of therapies.

**Supplementary Information:**

The online version contains supplementary material available at 10.1186/s40164-023-00403-4.

**To the editor**,

Acquired chronic idiopathic neutropenia (CIN) in adults is uncommon and its diagnosis can be asserted after exclusion of other etiologies [1]. Thus, the pathophysiologic classification of the underlying mechanisms of neutropenia can be challenging.

In autoimmune neutropenia (AIN), destruction of neutrophils or their precursors by anti-neutrophil autoantibodies (NA) is the hallmark of the disease [2]. However, immune attack by cytotoxic T lymphocytes (CTL), in conditions such as T large granular lymphocytic leukemia (T-LGL) can be also invoked [3–6]. In analogy to other forms of clonality (*e.g.,* monoclonal gammopathy of undetermined significance [MGUS], clonal hematopoiesis of indeterminate potential [CHIP]), T cell clonopathy of undetermined significance (TCUS) may exemplify an early stage of T-LGL, with neutropenia as a paraneoplastic epiphenomenon [3, 6]. The availability of NGS revealed another diagnostic consideration of neutropenia: clonal cytopenia of undetermined significance (CCUS) [7]. The exact mechanism by which CCUS can lead to neutropenia is not well understood, but T cell-mediated responses, directed to eliminate the abnormal myeloid clones, may play a role.

In a single center cohort, we studied 583 adult patients with unexplained neutropenia from 2000 to 2021 (Fig. 1A-B) and retrospectively identified 238 of them fulfilling the diagnostic criteria for chronic isolated neutropenia (Fig. 1 and Additional file 1: Figure S1) in order to analyze the associations with other conditions, discern its variable presentations, clinical course, and responsiveness to treatment. CIN was identified in the absence of any other known cause of neutropenia.

**Fig. 1:**
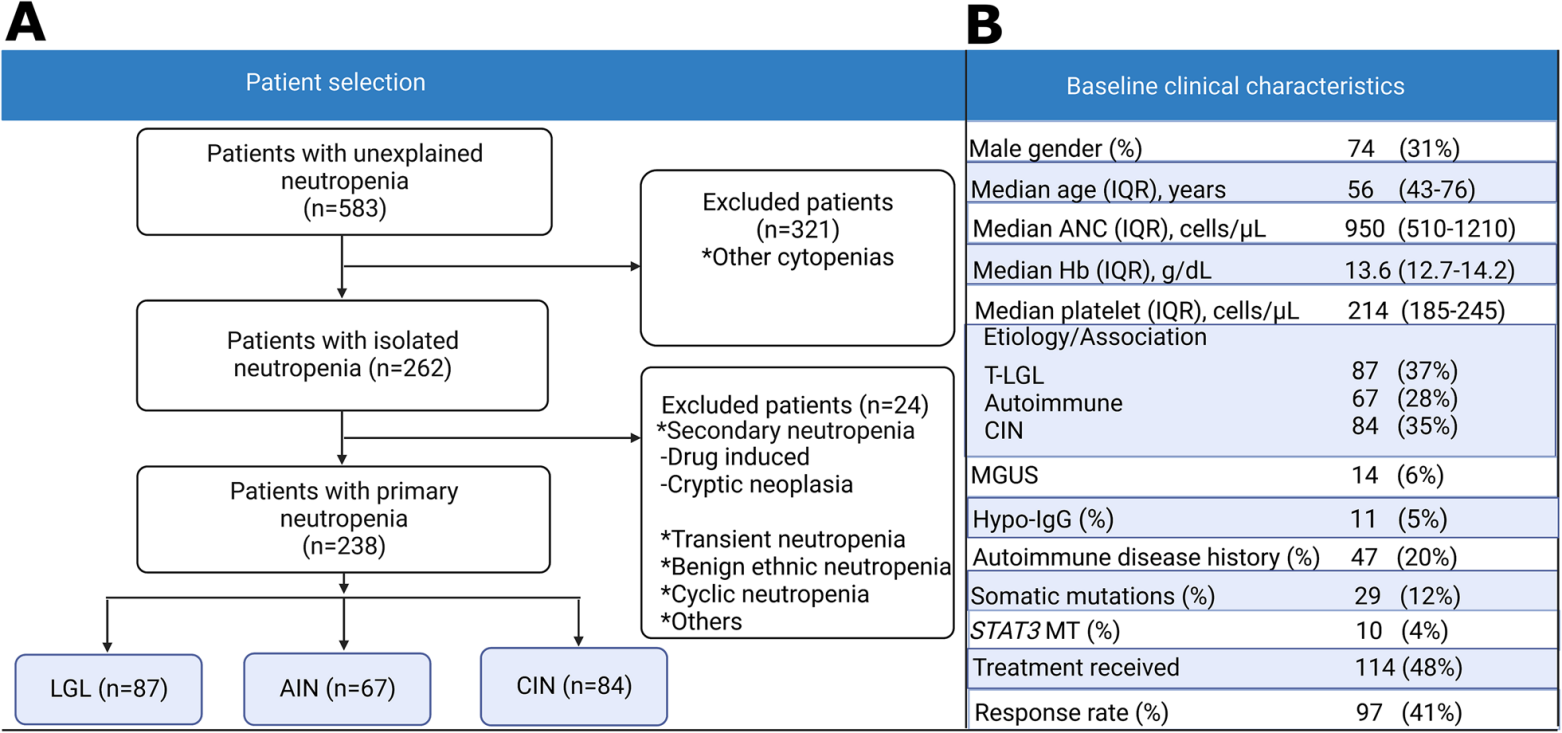
Chronic neutropenia in adults. **A** A flow diagram showing the selection of the patients included in our study cohort and exclusion criteria. Patients with other cytopenia (anemia and/or thrombocytopenia, n = 321) and patients with secondary neutropenia (n = 23) were excluded. **B** Clinical parameters and demographics of the cohort. *LGL* large granular leukemia, *AIN* autoimmune neutropenia, *MGUS* monoclonal gammopathy of undetermined significance, *Hypo-IgG* hypogammaglobulinemia, *CIN* chronic idiopathic neutropenia, *MT* mutation, *Hb* hemoglobin, *ANC* absolute neutrophil count, *IQR* interquartile range

We categorized patients according to the associated conditions including T-LGL (37%) and AIN (28%), whereas in 84 (35%) patients, the underlying cause of neutropenia was unexplained and hence referred to as CIN (Additional file 1: Table S1, Figure S1 for diagnostic criteria). Only 2% of patients exhibited T-LGL/AIN overlap. MGUS (6% overall) was present in 2% and 12% of AIN and T-LGL patients with CIN (Fig. 2A, upper and lower panels), whereas 5% of our cohort had hypogammaglobinemia. Notably, 17% of our cohort presented with splenomegaly.

**Fig. 2:**
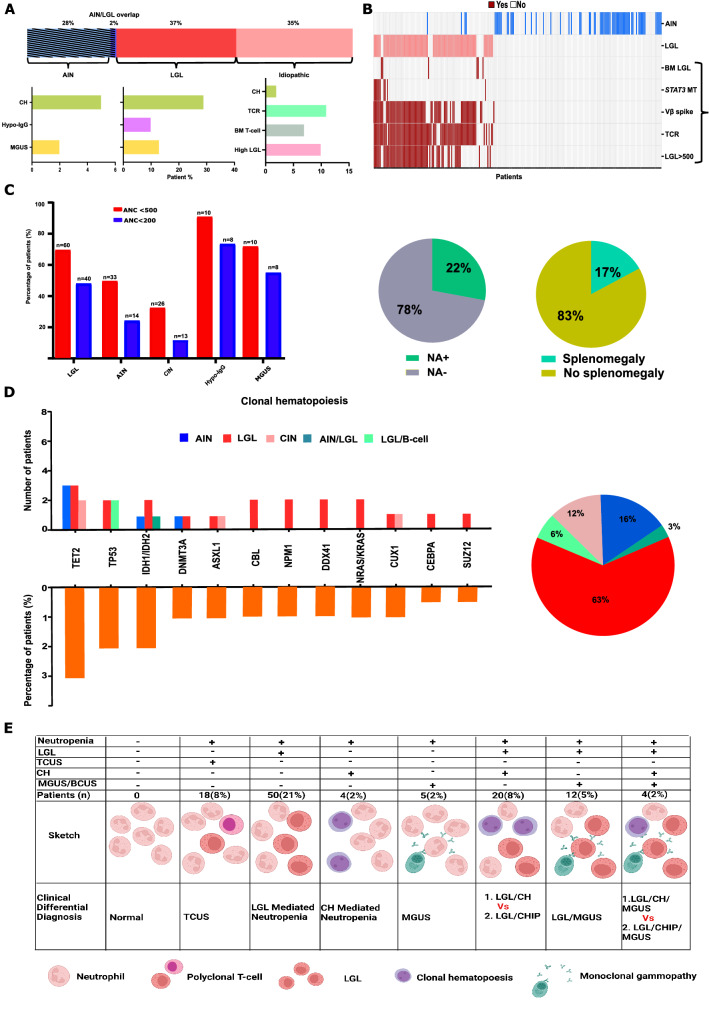
Overview of chronic neutropenia etiologies and clinical features in adults. **A** Bar graph showing the different etiologies of neutropenia, including overlap (upper panel). The lower panel is showing the percentages of CH, Hypo-IgG and MGUS among AIN and LGL cases. The bar graph below the idiopathic (pink) subgroup presents the percentage with high LGL count detected in this cohort. **B** Oncoplot of all the patients included in our study illustrating cases with AIN (blue), LGL (pink) and features suggestive of cytotoxic T-cell lymphocytes (CTL) including: Vbeta (Vβ) flow cytometry, T-cell receptor (TCR) polymerase chain reaction (PCR), absolute LGL count, bone marrow (BM) LGL infiltrate, and *STAT3* mutation (MT). **C** Bar histogram showing the percentage of patients with an absolute neutrophil count less than 200/μl (red) and less than 500/μl (blue) across each diagnostic subgroup. Pie charts showing percentage of patients with anti-neutrophil autoantibodies (NA) and splenomegaly among all patients with neutropenia (n = 238). **D** Histograms showing the number (upper panel) and the percentage (lower panel) of patients with gene mutations among different chronic neutropenia causes. The pie chart shows the percentages of different neutropenia causes among patients with gene mutations. **E** The table outlines all possible pathophysiological mechanisms of neutropenia including overlap causes. *LGL*: large granular leukemia, *CH* clonal hematopoiesis, *MGUS* monoclonal gammopathy of undetermined significance. *Hypo-IgG* hypogammaglobulinemia, *ANC* absolute neutrophils count, *AI* autoimmune, *ANA* anti-neutrophil antibody, *TCUS* T-cell clonality of undetermined significance, *BCUS* B-cell clonality of undetermined significance, *CHIP* clonal hematopoiesis of indeterminate potential

We identified patients (21% of CIN) with potential features of T-LGL not qualifying for the formal diagnosis of T-LGL, including cases with minor T-cell clones by Vβ flow cytometry (n = 18/84). Therefore, we referred to them as having TCUS (Additional file 1: Table S1). However, unlike T-LGL, none of these patients had *STAT3* mutation and in 50% of the cases, TCR rearrangement was not diagnostic, suggesting a clonally less polarized form of T-LGL (Additional file 1: Figure S2). Overall, 53% of our cohort had bone marrow biopsy. T-LGL and TCUS patients were found to have T-cell clones in the bone marrow. However, patients with AIN and CIN were not found to have any significant abnormalities or dysplastic features. The diagnostic features of T-LGL, AIN, and CIN are summarized in (Fig. 2B). Vβ- skewing and absolute T-LGL count were, as expected, less pronounced in CIN with TCUS than in T-LGL (Additional file 1: Figure S3). The proportion of patients with ANC < 200 cells/µL in our T-LGL, AIN, and CIN patients was 46%, 21%, and 15% (Fig. 2C).

CH was detected in 12% of patients including 4%, 28%, and 2% of cases with AIN, T-LGL, and CIN, respectively (Fig. 2A). The most frequently mutated gene was *TET2* (3% of the cases; Fig. 2D). While the presence of neutropenia and CH could fulfill the provisional diagnosis of CCUS, one cannot distinguish whether neutropenia is due to a CTL-mediated processes with coincidental CH, or indeed a true CCUS. Nevertheless, clinical combinations included: (i) LGL/CH (8%), wherein neutropenia could be either T cell-mediated with a subsequent gain of escape mutants or reflect T cell surveillance reaction to CH; (ii) T-LGL/MGUS (5%), wherein the neutropenia could potentially be T cell and/or B cell-mediated; (iii) T-LGL/CH/MGUS overlap (2%; Fig. 2E).

We then looked at response to different treatments as a possible surrogate of underlying mechanisms. A total of 114 patients received treatment (≥ 1 regimen) (Additional file 1: Figure S4). The most common indication for treatment was recurrent infections from severe neutropenia. Overall response rates at median follow up of 126 months (IQR 63–189) were compared with 35 studies (Additional file 1: Table S2) with CH patients showing no significant differences in response to therapy (OR 0.9, 95% CI 0.4–1.2).

Herein we have observed that a significant fraction of otherwise idiopathic cases appears to be often related to CTL-mediated processes as a part of a continuum starting from polyclonal responses, oligoclonality, TCUS, and culminating in a fully-blown T-LGL [4–6] (Fig. 2E and Additional file 1: Figure S5). Recently, the detection of CH has broadened the understanding of the potential roles of somatic mutations in neutropenia [7]. Here, the boundaries of nomenclature may be blurred as such patients may have CCUS and TCUS/asymptomatic T-LGL *vs.* TCUS with neutropenia and asymptomatic CH (*e.g.,* CHIP) depending on which process contributes more to the pathogenesis of neutropenia. A CH prevalence of 12% was significant compared to expected (2%) in aged –matched controls, given the median age of 56 years (p = 0.017) [8]. According to one unifying hypothesis, neutropenia in CCUS with a small clonal burden could be only explained by a CTL-mediated tumor surveillance reaction directed towards genetically aberrant CH clones [9].

In summary, diagnostic platforms allow for a more rational assessment of the pathogenesis and treatment of adult neutropenia [10]. Features compatible with a T cell process would point towards immunosuppressive therapy against T cells, AIN could be rationally targeted with anti B-cell therapies, while pure CCUS with a large clone would warrant workup for myelodysplasia. Further validating studies will be needed in the future to uncover the pathogenesis of isolated neutropenia, which will impact the treatment decisions.

## Supplementary Information

**Additional file 1: Table S1**. Definitions and nomenclatures. **Table S2**. Treatment and response rates in our cohort compared to previous studies in the literature.** Figure S1**. Spectrum of neutropenia in adults. After the exclusion of secondary causes of neutropenia, their intrinsic nature can be delineated based on etiology or association. The majority can be classified either as immune-mediated or idiopathic. The former includes antibody- and cell-mediated. The hallmark features and differential diagnosis are described. **Figure S2**. Conceptual figure demonstrating TCUS as a less polarized version of T-LGL. T-cell clones in TCUS are polyclonal in contrast to oligoclonal T-cell clones in LGL. **Figure S3**. Comparison between T-large granular lymphocytosis and T-cell clonality of undetermined significance.T-cell receptors Vβexpression. Bar histogram showing the Vβexpression in T-large granular lymphocytosispatientscompared to patients diagnosed with T-cell clonality of undetermined significance.Absolute large granular lymphocytes count. Bar histogram showing the absolute large granular lymphocytes count in T-large granular lymphocytic leukemiapatientscompared to patients diagnosed with T-cell clonality of undetermined significance. **Figure S4**. Neutropenia treatments and overall response rates. Bar histogram showing the percentage of different treatment used in our cohort. The shaded areas present the overall response rate. AI: autoimmune, LGL: large granular lymphocytosis, CH: clonal hematopoiesis, TCUS: T-cell clonality of undetermined significance, MMF: mycophenolate mofetil, ATG: anti-thymocyte globulin, IVIG: intravenous immunoglobulin. **Figure S5**. The role of clonal hematopoiesis in the pathophysiology of idiopathic neutropenia. Scenarios for the evolution of clonal hematopoiesis in neutropenia patients. LGL: large granular leukemia, CH: clonal hematopoiesis, TCUS: T-cell clonality of undetermined significance.

## Data Availability

Requests for datasets and materials not available in the main text or supplementary materials should be sent to the corresponding author: maciejj@ccf.org.
