# Supplementary material for: New approaches to idiopathic neutropenia in the era of clonal hematopoiesis
Source: Exp Hematol Oncol. 2023 Apr 28;12:42. doi: 10.1186/s40164-023-00403-4 (PMC10148514; doi:10.1186/s40164-023-00403-4)
Supplement: Supplementary file 1 — Additional file 1: Table S1. Definitions and nomenclatures. Table S2. Treatment and response rates in our cohort compared to previous studies in the literature. Figure S1. Spectrum of neutropenia in adults. After the exclusion of secondary causes of neutropenia, their intrinsic nature can be delineated based on etiology or association. The majority can be classified either as immune-mediated or idiopathic. The former includes antibody- and cell-mediated. The hallmark features and differential diagnosis are described. Figure S2. Conceptual figure demonstrating TCUS as a less polarized version of T-LGL. T-cell clones in TCUS are polyclonal in contrast to oligoclonal T-cell clones in LGL. Figure S3. Comparison between T-large granular lymphocytosis and T-cell clonality of undetermined significance.T-cell receptors Vβexpression. Bar histogram showing the Vβexpression in T-large granular lymphocytosispatientscompared to patients diagnosed with T-cell clonality of undetermined significance.Absolute large granular lymphocytes count. Bar histogram showing the absolute large granular lymphocytes count in T-large granular lymphocytic leukemiapatientscompared to patients diagnosed with T-cell clonality of undetermined significance. Figure S4. Neutropenia treatments and overall response rates. Bar histogram showing the percentage of different treatment used in our cohort. The shaded areas present the overall response rate. AI: autoimmune, LGL: large granular lymphocytosis, CH: clonal hematopoiesis, TCUS: T-cell clonality of undetermined significance, MMF: mycophenolate mofetil, ATG: anti-thymocyte globulin, IVIG: intravenous immunoglobulin. Figure S5. The role of clonal hematopoiesis in the pathophysiology of idiopathic neutropenia. Scenarios for the evolution of clonal hematopoiesis in neutropenia patients. LGL: large granular leukemia, CH: clonal hematopoiesis, TCUS: T-cell clonality of undetermined significance. [file 40164_2023_403_MOESM1_ESM.docx]

**Additional files**

**New approaches to idiopathic neutropenia in the era of clonal hematopoiesis**

**Additional tables:** 2

**Additional figures:** 5

**References:** 28

**Table S1. Definitions and nomenclatures**

| Term | Abbreviation | Definition |
| --- | --- | --- |
| Chronic idiopathic neutropenia | CIN | Chronic neutropenia for more than 4 months in the absence of known etiology. |
| Autoimmune neutropenia | AIN | Chronic neutropenia secondary to anti-neutrophil antibodies against neutrophil or neutrophil precursors. |
| T-cell large granular lymphocytic leukemia | T-LGL | Clonal expansion of T-cells in the peripheral blood or bone marrow^1^. |
| T-cell clonopathy of undetermined significance | T-CUS | Oligo/poly-clonal expansion of T-cells that does not fulfil the diagnostic criteria of T-LGL^2^ |
| Cytotoxic T-cell mediated neutropenia | CTL | Chronic neutropenia secondary to T-cell mediated process (e.g., T-LGL or T-CUS). |
| Clonal hematopoiesis of indeterminate potential | CHIP | Single or multiple myeloid mutations in the peripheral blood or bone marrow in the absence of myeloid neoplasia or attributable cytopenia^3^ |
| Clonal cytopenia of undetermined significance | CCUS | Neutropenia (in our study) secondary to single or multiple somatic myeloid mutations, with allelic burden more than 2%, in the peripheral blood and/or bone marrow in the absence of all other known causes of neutropenia^3^ |
| Monoclonal gammopathy of undetermined significance | MGUS | Pre-neoplastic plasma cell disorder that is characterized by serum M-protein less than 30 g/L, bone marrow clonal plasma cells less than 10 percent, and absence of plasma cell myeloma-related end-organ damage ^4^ |

**Table S2. Treatment and response rates in our cohort compared to previous studies in the literature**

| Neutropenia etiology | Treatment | Our cohort  number treated | Our Cohort  response rate (%) | Response rate (%) in prior studies |
| --- | --- | --- | --- | --- |
| AIN | Campath | 1 | 100% | 75%^5^ |
|  | Rituximab | 7 | 42.9% | 25%^6^ |
|  | IVIG | 2 | 50% | 50%^7^ |
|  | Growth factors | 13 | 84.6% | 90%^7,8^ |
|  | Steroids | 15 | 60% | 75%^9^ |
| CIN+AIN | IST (steroids, cyclophosphamide, methotrexate) | 33 | 45.5% | 42%^9^ |
|  | Growth factors | 21 | 90.5% | 86%^9^ |
| Felty’s syndrome | Growth factors | 1 | 100% | 100%^10^ |
|  | Splenectomy | 0 |  | 67%^11^, 88%^12^, 20%^13^ |
|  | Steroids | 0 |  | 13%^14^ |
| T-cell mediated | Steroids | 21 | 66.7% | 60%^15^, 63%^13^, 9%^16^, 73%^17^ |
|  | Methotrexate | 35 | 43% | 75%^13^ ,55%^16^, 88%^18^, 100%^19^ |
|  | Cyclophosphamide | 22 | 36.4% | 66%^16^, 25%^18^, 69%^17^ |
|  | Cyclosporine | 35 | 40% | 21%^16^, 78%^18^, 33%^20^, 100%^21^, 56%^22^ |
|  | Splenectomy | 7 | 42.9% | 31%^16^, 0%^18,23^, 63%^17^, 50%^24^ |
|  | Growth factors | 33 | 84.8% | 83%^18^ |
| CIN | Steroids | 9 | 33.3% | 100%^25^ |

AIN: autoimmune neutropenia, CIN: chronic idiopathic neutropenia, LGL: large granular leukemia, IVIG: intravenous immunoglobulin, IST: immunosuppressive therapy

**Figure S1**


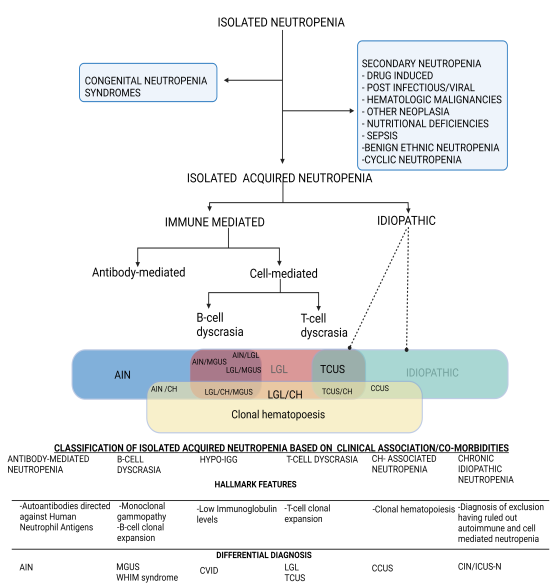


**Figure S2**


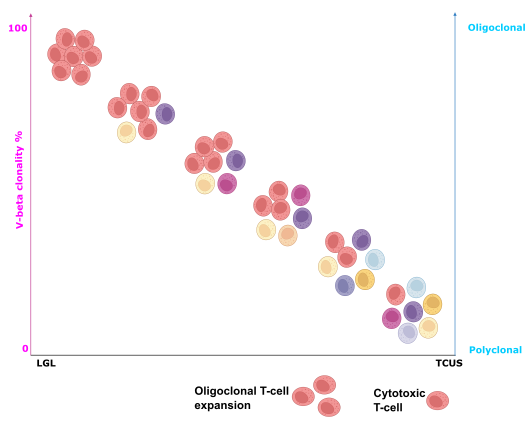
 **Figure S3**


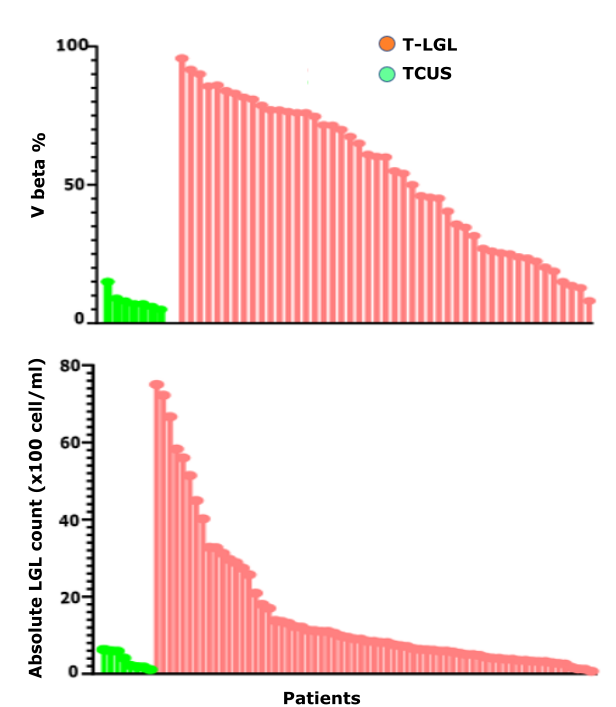


**Figure S4**


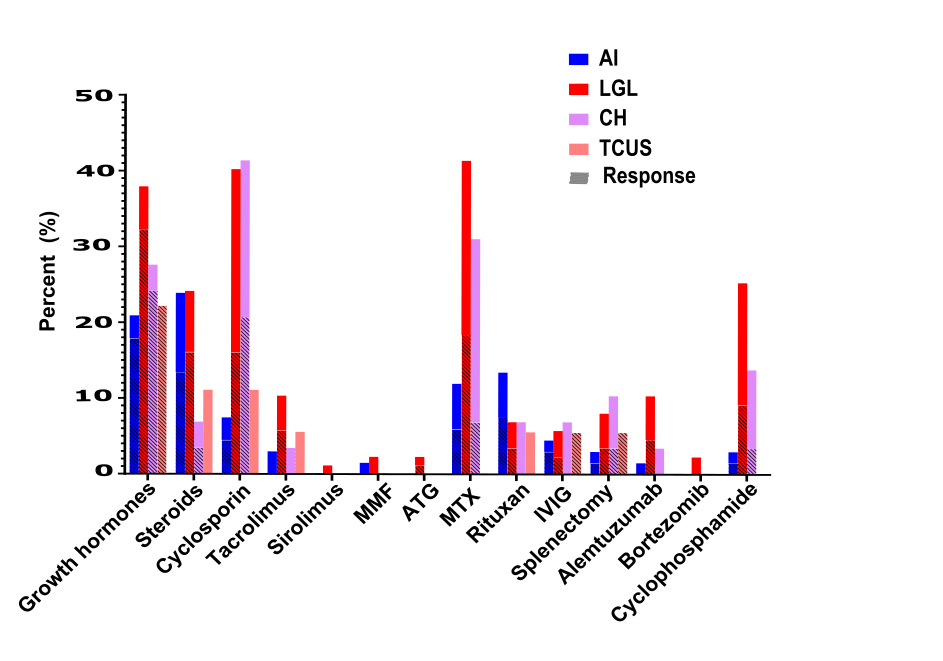


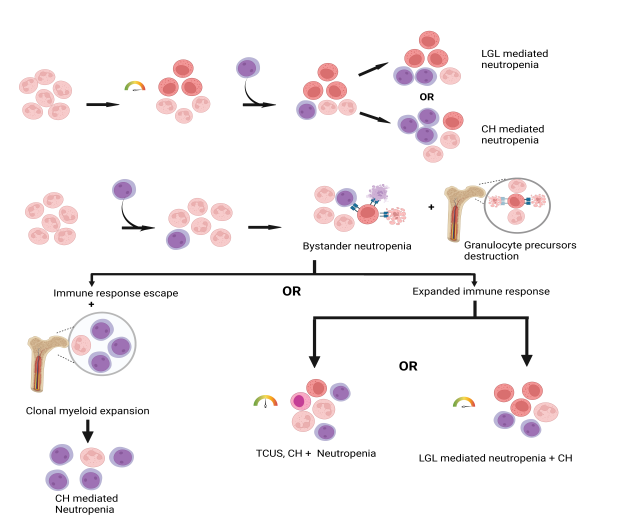
**Figure S5**

**Additional Methods**

**Genetic studies**

Molecular data were collected from a number of targeted sequencing panels (TruSeq, Nextera, and diagnostic NGS). Sequencing libraries were generated according to an Illumina paired-end library protocol. The enriched targets were sequenced using a HiSeq 2000 or MiSeq (Illumina), at 862x coverage. Variants were annotated using Annovar14 and filtered by removing: i) synonymous single nucleotide variants; ii) variants only present in 140 unidirectional reads; and iii) variants in repetitive genomic regions. Variants with minimum depth less than 20 or number of high-quality reads less than 5 were filtered out. A bio-analytic pipeline developed in-house^26,27^ identified somatic mutations using sequences derived from controls and mutational databases such as dbSNP138, 1000 Genomes or ESP 6500 database, and Exome Aggregation Consortium (ExAC). Variant allelic frequencies (VAFs) were adjusted according to zygosity and copy number based on conventional metaphase karyotyping and/or single nucleotide polymorphism array results.

**Flow cytometry**

Fresh peripheral blood samples were stained with a panel of antibodies for V-beta (Vβ) analysis to quantitate the percentage of each Vβ family in CD4 and CD8 lymphocytes, as previously described by our group^28^. Flow cytometric evidence of an abnormal CTL population was based on the expression of CD2, CD3, TCRαβ (or γδ), CD4, CD5dim, CD8, CD16/56, or CD57 with negativity of CD28. In addition, TCR Vβ expansions were detected and quantitated by flow cytometry according to criteria previously described^28^.

**Polymerase chain reaction (PCR)**

RNA isolation and complementary DNA synthesis, CDR3 region amplification, CDR3 size analysis, CDR3 cloning, spectratyping, sequencing, and subsequent analysis of “clone size” were performed as previously described^28^.

**Additional figure legends**

**Figure S1. Spectrum of neutropenia in adults.** After the exclusion of secondary causes of neutropenia, their intrinsic nature can be delineated based on etiology or association. The majority can be classified either as immune-mediated or idiopathic. The former includes antibody- and cell-mediated. The hallmark features and differential diagnosis are described.

**Figure S2.** Conceptual figure demonstrating TCUS as a less polarized version of T-LGL. T-cell clones in TCUS are polyclonal in contrast to oligoclonal T-cell clones in LGL.

**Figure S3. Comparison between T-large granular lymphocytosis and T-cell clonality of undetermined significance.** **(A)** T-cell receptors Vβexpression. Bar histogram showing the Vβexpression in T-large granular lymphocytosis (T-LGL) patients (pink) compared to patients diagnosed with T-cell clonality of undetermined significance (green). **(B)** Absolute large granular lymphocytes count. Bar histogram showing the absolute large granular lymphocytes count in T-large granular lymphocytic leukemia (T-LGL) patients (pink) compared to patients diagnosed with T-cell clonality of undetermined significance (green).

**Figure S4. Neutropenia treatments and overall response rates.** Bar histogram showing the percentage of different treatment used in our cohort. The shaded areas present the overall response rate. AI: autoimmune, LGL: large granular lymphocytosis, CH: clonal hematopoiesis, TCUS: T-cell clonality of undetermined significance, MMF: mycophenolate mofetil, ATG: anti-thymocyte globulin, IVIG: intravenous immunoglobulin.

**Figure S5: The role of clonal hematopoiesis in the pathophysiology of idiopathic neutropenia.** Scenarios for the evolution of clonal hematopoiesis in neutropenia patients. LGL: large granular leukemia, CH: clonal hematopoiesis, TCUS: T-cell clonality of undetermined significance.

**References**

1. Lamy T, Moignet A, Loughran TP, Jr. LGL leukemia: from pathogenesis to treatment. Blood 2017;129:1082-94.

2. Dippel E, Klemke D, Hummel M, Stein H, Goerdt S. T-cell clonality of undetermined significance. Blood 2001;98:247-8.

3. DeZern AE, Malcovati L, Ebert BL. CHIP, CCUS, and Other Acronyms: Definition, Implications, and Impact on Practice. Am Soc Clin Oncol Educ Book 2019;39:400-10.

4. Blade J. Clinical practice. Monoclonal gammopathy of undetermined significance. N Engl J Med 2006;355:2765-70.

5. Marsh JC, Gordon-Smith EC. CAMPATH-1H in the treatment of autoimmune cytopenias. Cytotherapy 2001;3:189-95.

6. Dungarwalla M, Marsh JC, Tooze JA, et al. Lack of clinical efficacy of rituximab in the treatment of autoimmune neutropenia and pure red cell aplasia: implications for their pathophysiology. Ann Hematol 2007;86:191-7.

7. Bux J, Kissel K, Nowak K, Spengel U, Mueller-Eckhardt C. Autoimmune neutropenia: clinical and laboratory studies in 143 patients. Ann Hematol 1991;63:249-52.

8. Dale DC, Bonilla MA, Davis MW, et al. A randomized controlled phase III trial of recombinant human granulocyte colony-stimulating factor (filgrastim) for treatment of severe chronic neutropenia. Blood 1993;81:2496-502.

9. Sicre de Fontbrune F, Moignet A, Beaupain B, et al. Severe chronic primary neutropenia in adults: report on a series of 108 patients. Blood 2015;126:1643-50.

10. Stanworth SJ, Bhavnani M, Chattopadhya C, Miller H, Swinson DR. Treatment of Felty's syndrome with the haemopoietic growth factor granulocyte colony-stimulating factor (G-CSF). QJM 1998;91:49-56.

11. Blumfelder TM, Logue GL, Shimm DS. Felty's syndrome: effects of splenectomy upon granulocyte count and granulocyte-associated IgG. Ann Intern Med 1981;94:623-8.

12. Laszlo J, Jones R, Silberman HR, Banks PM. Splenectomy for Felty's syndrome. Clinicopathological study of 27 patients. Arch Intern Med 1978;138:597-602.

13. Loughran TP, Jr., Kidd PG, Starkebaum G. Treatment of large granular lymphocyte leukemia with oral low-dose methotrexate. Blood 1994;84:2164-70.

14. Barnes CG, Turnbull AL, Vernon-Roberts B. Felty's syndrome. A clinical and pathological survey of 21 patients and their response to treatment. Ann Rheum Dis 1971;30:359-74.

15. Loughran TP, Jr., Starkebaum G. Large granular lymphocyte leukemia. Report of 38 cases and review of the literature. Medicine (Baltimore) 1987;66:397-405.

16. Bareau B, Rey J, Hamidou M, et al. Analysis of a French cohort of patients with large granular lymphocyte leukemia: a report on 229 cases. Haematologica 2010;95:1534-41.

17. Dhodapkar MV, Li CY, Lust JA, Tefferi A, Phyliky RL. Clinical spectrum of clonal proliferations of T-large granular lymphocytes: a T-cell clonopathy of undetermined significance? Blood 1994;84:1620-7.

18. Osuji N, Matutes E, Tjonnfjord G, et al. T-cell large granular lymphocyte leukemia: A report on the treatment of 29 patients and a review of the literature. Cancer 2006;107:570-8.

19. Hamidou M, Lamy T. [Large granular lymphocyte proliferations. Clinical and pathogenic aspects]. Rev Med Interne 2001;22:452-9.

20. Fortune AF, Kelly K, Sargent J, et al. Large granular lymphocyte leukemia: natural history and response to treatment. Leuk Lymphoma 2010;51:839-45.

21. Sood R, Stewart CC, Aplan PD, et al. Neutropenia associated with T-cell large granular lymphocyte leukemia: long-term response to cyclosporine therapy despite persistence of abnormal cells. Blood 1998;91:3372-8.

22. Battiwalla M, Melenhorst J, Saunthararajah Y, et al. HLA-DR4 predicts haematological response to cyclosporine in T-large granular lymphocyte lymphoproliferative disorders. Br J Haematol 2003;123:449-53.

23. Newland AC, Catovsky D, Linch D, et al. Chronic T cell lymphocytosis: a review of 21 cases. Br J Haematol 1984;58:433-46.

24. Loughran TP, Jr., Starkebaum G, Clark E, Wallace P, Kadin ME. Evaluation of splenectomy in large granular lymphocyte leukaemia. Br J Haematol 1987;67:135-40.

25. Dale DC, Guerry Dt, Wewerka JR, Bull JM, Chusid MJ. Chronic neutropenia. Medicine (Baltimore) 1979;58:128-44.

26. Makishima H, Yoshizato T, Yoshida K, et al. Dynamics of clonal evolution in myelodysplastic syndromes. *Nat Genet*. 2017;49(2):204-212. doi:10.1038/ng.3742.

27. Hirsch CM, Nazha A, Kneen K, et al. Consequences of mutant TET2 on clonality and subclonal hierarchy. *Leukemia*. 2018;32(8):1751-1761. doi:10.1038/s41375-018-0150-9

28. Clemente MJ, Przychodzen B, Jerez A, et al. Deep sequencing of the T-cell receptor repertoire in CD8+ T-large granular lymphocyte leukemia identifies signature landscapes. Blood 2013;122:4077-85.
